# Ab locks for improving the selectivity and safety of antibody drugs

**DOI:** 10.1186/s12929-020-00652-z

**Published:** 2020-06-25

**Authors:** Wen-Wei Lin, Yun-Chi Lu, Chih-Hung Chuang, Tian-Lu Cheng

**Affiliations:** 1 Department of Laboratory Medicine, School of Medicine, College of Medicine, Kaohsiung Medical University, Kaohsiung, Taiwan; 2 Graduate Institute of Medicine, College of Medicine, Kaohsiung Medical University, Kaohsiung, Taiwan; 3 Drug Development and Value Creation Research Center, Kaohsiung Medical University, Kaohsiung, Taiwan; 4 Department of Medical Research, Kaohsiung Medical University Hospital, Kaohsiung, Taiwan; 5 Department of Biomedical and Environmental Biology, Kaohsiung Medical University, 100 Shih-Chuan 1st Road, Kaohsiung, 80708 Taiwan; 6 Department of Medical Laboratory Science and Biotechnology, College of Health Sciences, Kaohsiung Medical University, Kaohsiung, Taiwan

**Keywords:** Monoclonal antibody (mAb), adverse events, Ab lock, spatial-hindrance-based approaches, affinity-based approaches

## Abstract

Monoclonal antibodies (mAbs) are a major targeted therapy for malignancies, infectious diseases, autoimmune diseases, transplant rejection and chronic inflammatory diseases due to their antigen specificity and longer half-life than conventional drugs. However, long-term systemic antigen neutralization by mAbs may cause severe adverse events. Improving the selectivity of mAbs to distinguish target antigens at the disease site from normal healthy tissue and reducing severe adverse events caused by the mechanisms-of-action of mAbs is still a pressing need. Development of pro-antibodies (pro-Abs) by installing a protease-cleavable Ab lock is a novel and advanced recombinant Ab-based strategy that efficiently masks the antigen binding ability of mAbs in the normal state and selectively “turns on” the mAb activity when the pro-Ab reaches the proteolytic protease-overexpressed diseased tissue. In this review, we discuss the design and advantages/disadvantages of different Ab lock strategies, focusing particularly on spatial-hindrance-based and affinity peptide-based approaches. We expect that the development of different masking strategies for mAbs will benefit the local reactivity of mAbs at the disease site, increase the therapeutic efficacy and safety of long-term treatment with mAbs in chronic diseases and even permit scientists to develop Ab drugs for formerly undruggable targets and satisfy the unmet medical needs of mAb therapy.

## Background

Monoclonal antibodies (mAbs) exist naturally in the human body and have become a mainstream therapeutic option for several kinds of diseases in the clinic, such as autoimmune diseases [24], infectious diseases [22], malignancies [152] and transplant rejection [95]. The pharmaceutical industry has shown continued interest in developing mAb products. This interest is partially driven by the high specificity of mAbs to target antigens as compared with small molecular drugs, their long half-life during systemic circulation, and a well-established, cost-effective platform for producing mAbs to improve the yields, reduce the manufacturing cost and minimize the unexpected safety issues in clinical trials [42, 94, 98, 132, 191]. The first therapeutic mAb, Muromonab-CD3 (also known as Orthoclone OKT3) was a mouse mAb that specifically targets human cluster of differentiation 3 (CD3) on T lymphocytes. It was approved by US Food and Drug Administration (FDA) for treating kidney transplant rejection in 1986. Since then, global sales and the approval rate of mAbs have shown dramatic growth annual (about four products per year). As of December 2019, 79 mAb products had been approved and marketed in the US and Europe and over 300 mAb products were in development [42, 99]. The market for mAbs is expected to continue to grow at a compound annual growth rate (CAGR) of 8% or more annually [42] and worldwide sales of mAb products are estimated to be nearly 300 billion US dollars by 2025 [99]. These figures suggest that mAbs will continue to play a dominant role as a major class of biopharmaceutical products worldwide. However, systemic neutralization of antigens by mAb drug administration carries the risk of a range of adverse events that are associated with the specific target antigens or mechanisms which are essential for the physiological behavior of normal tissues [61, 175].

Systemic administration of mAb drugs may induce severe adverse events by mechanism-of-action-related effects, which means that the mAb drugs eliminate target antigens that maintain physiological functions in normal tissues [61]. For example, in the field of autoimmune disease, mAbs such as Infliximab (Remicade®; Janssen Biotech) or Adalimumab (Humira®; Abbott), work directly against the pro-inflammatory cytokine, tumor necrosis factor α (TNF-α) to treat severe rheumatoid arthritis (RA) [44, 115, 172, 175]. Due to the key role of TNF-α in *Mycobacterium tuberculosis* (*M. tuberculosis*) infection and immunity [8, 77], anti-TNF-α therapy increases the frequency of latent tuberculosis reactivation and is also associated with an increased risk of other serious infections and malignancies [41, 140]. It has been reported that progressive multifocal leukoencephalopathy (PML), which is a fatal and rapidly progressive demyelinating disease, has been induced by reactivating latent infection with the polyomavirus John Cunningham virus (JCV) in the central nervous system after treatment with Natalizumab (Tysabri®; Biogen). Natalizumab is a humanized anti-adhesion molecule α4 integrin mAb that is used to treat multiple sclerosis by directly combating T cell trafficking and adhesion [97, 101, 135, 143]. Based on clinical trial data from a 3,147 patient cohort study, the risk frequency of PML corresponds to about 1 to 1000 patients after 18 months of Natalizumab treatment [195] and may originate from immunosuppression of T cell depletion. A similar situation was observed in the field of oncology, when Rituximab (Rituxan®; Genentech), a chimeric anti-CD20 mAb, was used to treat non-Hodgkin’s lymphoma (NHL) by directly eliminating of B cells. The NHL patients receiving Rituximab therapy were reported to have decreased host immunity that triggered PML disease by reactivating JCV [4, 19]. The humanized mAb against CD11a (Efalizumab; Reptiva®; Genentech) was reported to be associated with four PML cases during chronic plaque psoriasis treatment. Suspension of marketing authorization was recommended by the European Medicines Agency (EMA) and phased voluntary withdrawal took place in the United States [20, 114]. Alemtuzumab (Lemtrada®; Campath®; Genzyme), is a humanized anti-CD52 mAb used to avoid immune rejection by bone-marrow [23, 60] and renal [182] transplantation or treat chronic lymphocytic leukaemia (CLL) [91] and multiple sclerosis [67] by depleting CD52-expressing cells, such as CD4^+^ and CD8^+^ T cells, monocytes and nature killer cells (NK cells). However, around 3% patients with early multiple sclerosis treated with Alemtuzumab developed serious and fatal thrombocytopaenia [59, 63, 67] and 45% (5 of 11 patients) of patients with peripheral T cell lymphoproliferative disorders showed severe multi-lineage haematopoietic toxicity including lymphopaenia, neutropaenia and thrombocytopaenia [55]. Ipilimumab (Yervoy®; Bristol-Myers Squibb) is a mAb which specifically targets cytotoxic T-lymphocyte-antigen 4 (CTLA-4), one of the major immune checkpoints that regulate adaptive immune responses [47]. It is used to treat metastatic melanoma as a monotherapeutic agent and treat advanced renal cell carcinoma combined with Nivolumab (anti-PD-1 Ab; Opdivo®; Bristol-Myers Squibb) [74]. The CTLA-4 blockage can increase T cell stimulation and activity to continually attack tumor cells [103], but it often causes a range of immune-related adverse events such as rash, hepatitis and even enterocolitis that sometimes requires a colectomy [128, 183]. Humanized anti-HER2/neu mAb (Trastuzumab; Herceptin®; Genentech), used to treat HER2-positive metastatic breast cancer has been reported to possibly also target HER2-expressing cardiomyocytes, block all downstream signaling from HER2 and trigger congestive heart failure risk [170] or cardiac dysfunction in up to 4% patients with Transtuzumab monotherapy with higher incidence in patients receiving additional chemotherapy, such as anthracyclines [78, 129]. In addition, mAbs targeting immune cells (e.g., T or B cells), such as CD3-specific (Muromonab-CD3 )[130], CD20-specific (Rituximab) [188], CD28-specific (TGN1412) [165] or CD52-specific (Alemtuzumab) [186, 187] mAbs, cannot only deplete these targeted cells, but possibly also trigger immediate life-threating cytokine storm (also known as cytokine-released syndrome (CRS)) [29, 79, 135, 165, 185], causing a systemic inflammation response, organ injury and failure even leading to death [165]. As the systemic depletion of antigens may cause unpredictable reactions in patients during Ab therapy, we also list several side effects that have been documented for the above mentioned mAbs (Table 1). Improvement of the selectivity of mAb to distinguish target antigens or cells at the disease site from normal healthy tissue may improve safety and therapeutic efficacy during mAb therapy.

**Table 1:** Side effects of monoclonal Ab drugs caused by systemic on-target toxicity

mAb (Brand name; Company)	Target	Physiological distribution of target antigens	Indications	Selected adverse events	Refs
Adalimumab (Humira®; Abbott)/Infliximab (Remicade®; Janssen Biotech)	TNF-α	Activated macrophages, CD4^+^ T cells, NK cells, neutrophils, mast cells, eosinophils, and neurons	• Juvenilc idiopathic arthritis • Crohn’s disease • Ulcerative colitis • Rheumatoid arthritis • Ankylosing spondylitis • Psoriatic arthritis • Plaque psoriasis	• Anti-drug Ab (5%) • Increased nuclear-specific antibodies (12%) • Infections (e.g. 17% upper respiratory infection, increase 2-fold risk of tuberculosis) • Increase 2-3-fold risk of malignancies (e.g. lymphoma and lymphoproliferative disorders) • Anaemia, leukopaenia and thrombocytopaenia • Immunosuppression	[111, 145, 146]
Natalizumab (Tysabri®; Biogen)	α4 integrin	Activated endothelial cells, CD8^+^ T cells, leukocytes	• Multiple sclerosis	• Infusion and hypersensitivity reactions (6%) • Immunogenicity (6%) • Progressive multifocal leukoencephalopathy (PML) (0.4%) • Immunosuppression • Hepatotoxicity	[18, 54, 81, 181]
Rituximab (Rituxan®; Genentech)	CD20	B cells	• Non-Hodgkin’s Lymphoma (NHL) • Chronic Lymphocytic Leukemia (CLL) • Rheumatoid arthritis • Wegener’s Granulomatosis) • Microscopic Polyangiitis (MPA)	• Infection (20%) • Hepatitis B reactivation (8.7%) • Immunosuppression • Immunogenicity • Renal toxicity • CRS • PML	[51, 171]
Efalizumab (Reptiva®; Genentech)	CD11a	B and T cells, monocytes, macrophages, neutrophils, basophils, and eosinophils	• Plaque psoriasis	• Malignancies (1.8%) • Immune thrombocytopaenia (0.3%) • Guillain–Barré syndrome, encephalitis, meningitis • Immunosuppression • Immune haemolytic anaemia • PML	[6, 66, 147]
Alemtuzumab (Lemtrada®/Campath®; Genzyme)	CD52	B and T cells, monocytes, dendritic cells and mature sperm cells	• Multiple sclerosis • CLL • Kidney Transplantation	• Thyroid disorders (35%) • Thrombocytopaenia (2%) • Autoimmune neutropenia, haemolytic anaemia and autoimmune kidney diseases (0.3%) • Lymphoproliferative disorders • CRS	[13, 81, 137, 181]
Ipilimumab (Yervoy®; Bristol-Myers Squibb)	CTLA-4	Activated T cells, Treg	• Malignant Melanoma • Renal Cell Carcinoma • Metastatic Colorectal Cancer	• Colitis (11.1%) • Severe skin reaction (6.5%) • Hormone gland problems (especially the pituitary, adrenal, and thyroid glands)(4.6%) • Enterocolitis (2.1%) • Hepatitis (1.3%) • Pneumonitis (1.22%)	[169]
Trastuzumab (Herceptin®; Genentech)	HER2	Cells derived from all three germ layer (e.g. epithelial cells, cardiomyocytes … )	• Metastatic Breast Cancer • Metastatic Gastric Cancer	• Heart failure (2~7%) • Cardiotoxicity (2.6~4.5%) • Peripheral edema (5~10%) • Hypertension (4%) • Arrhythmia and palpitation (3%)	[30, 65, 69, 73, 85, 102, 116, 161]
Muromonab-CD3 (Orthoclone OKT3®; Centocor Ortho Biotech Products LP.)	CD3	CD4^+^ and CD8^+^ T cells	• Acute allograft rejection • Acute graft-versus-host disease (GVHD)	• Flu-like syndrome (50%) • Immunogenicity (3~61%) • Central nervous system complications (3%) • Immunosuppression and infections • CRS	[37, 154]

*TNF-α* tumor necrosis factor α, *CD* cluster of differentiation, *CTLA-4* cytotoxic T-lymphocyte-associated protein 4, *HER2* human epidermal growth factor receptor 2, *NK* natural killer cell, *Treg* regulatory T cell, *PML* progressive multifocal leukoencephalopathy, *CRS* cytokine-released syndrome

### Different masking strategies for pro-antibody drug development

**Fig. 1:**
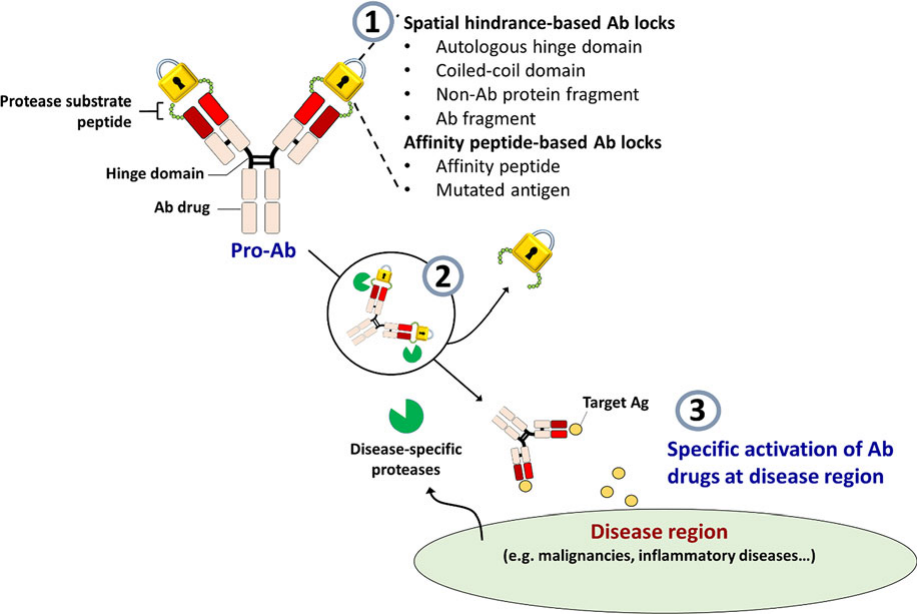
Schematic of pro-antibody selectively activated at the disease region by installing a protease-cleavable Ab lock. (1) Generation of pro-antibody (pro-Ab) by installing a protease-cleavable Ab lock is a novel and advanced recombinant Ab-based strategy that (2) selectively “turns on” the mAb activity when the pro-Ab reaches proteolytic enzyme (i.e., protease)-overexpressed diseased tissue, (3) locally neutralizing the target antigen and reducing on-target toxicity caused by systemic administration of Ab drugs during disease treatment. Ab, antibody; Ag, antigen

In order to increase the selectivity of mAbs at the disease site to allow them to carry out their function locally, mAb drugs should ignore the target antigen in normal healthy tissue and be preferentially active in the disease region. One way to achieve this goal is by generation of a pro-antibody (pro-Ab) by installing a protease-cleavable Ab lock, which was defined as the molecule that can interfere the antigen binding ability of Ab drugs, is a novel and advanced recombinant Ab-based strategy that selectively “turns on” mAb activity when the pro-Ab reaches proteolytic enzyme (i.e. protease)-overexpressed diseased tissue. A pro-Ab is made up of two essential parts, a “masking domain” that can physically block or interfere with the antigen binding ability of an mAb; and a substrate peptide of disease-associated proteases that connect the masking domain to the N-terminal of the light chain and/or heavy chain of the mAb. The addition of the masking domain results in mAbs with significantly reduced binding ability for their target antigens that, upon exposure to overexpressed proteases at disease site, reactivate the original mAb binding activity, thereby improving the selectivity of the mAb and preventing on-target toxicity during systemic circulation of mAb drugs (Fig. 1). In this review, we will discuss a variety of masking strategies (Table 2), especially spatial hindrance-based (i.e., mask antigen binding ability of mAb by sterically interference) and affinity peptide-based theory (i.e., occupation of an antigen binding site of a mAb by an affinity peptide) in the designing pro-Ab drugs, and discuss their various advantages and disadvantages.

**Table 2:** Different masking theories of Ab locks

Ab lock (MW)	Masking theory	Example Ab (Refs)	Schematic
**Spatial hindrance-based Ab lock**			
Autologous hinge domain (3.5 kDa)	Human IgG1 hinge forms disulfide bond for sterically interfering with the antigen binding ability of Ab drugs	• Infliximab (anti-TNF-α mAb) [100]	
Coiled-coil (CC) domain (5 ~ 9.2 kDa)	Covalent and non-covalent CC domains and helix-turn-helix domains derived from de novo designs or native human proteins (e.g. c-Fos and c-Jun) can form a robust secondary structure and sterically block the CDRs of Ab drugs from binding antigen	• anti-CD19 Ab (clone hBU12) [176]	
		• Rituximab (anti-CD20 mAb) [176]	
		• Trastuzumab (anti-HER2 mAb) [176]	
		• h15H3 (anti-αVβ6 mAb) [176]	
		• 145-2C11 (anti-mouse CD3 mAb) [176]	
Non-Ab protein fragment (40 kDa)	Latency-associated peptide (LAP) derived from transforming growth factor-β (TGF-β) sterically interferes the antigen binding ability of Ab drugs	• Cetuximab (anti-EGFR Ab) [27]	
		• Infliximab [27]	
Ab fragment (26 kDa)	The outer disulfide-stabilized variable fragment (dsFv) or whole Ab, which against specific antigen, can shield the inner antigen binding domain of another Ab drug	• anti-c-Met dsFv [108]	
		• Infliximab • Adalimumab (anti-TNF-α mAb) [124]	
		• anti-CTLA-4 Ab (clone 24H2) [125]	
**Affinity peptide-based Ab lock**			
Affinity peptide (2.8 ~ 5 kDa)	Binding peptide from bacterial peptide display library that could specifically occupy the antigen-binding site of Ab drug	• Cetuximab [35]	
		• anti-VCAM-1 Ab [43]	
		• Panitumumab (anti-EGFR mAb) [193]	
		• Panitumumab-DM1 [93, 192]	
		• anti-HIV p17 Ab [70]	
Mutated antigen (23.3 kDa)	Point mutated soluble EGFR domain III (sEGFRdIII) can be bound by anti-EGFR Ab, thereby masking its antigen binding ability in the condition without protease presentation	• Cetuximab [36] • Matuzumab (anti-EGFR mAb) [36]	

*Ab* antibody, *IgG1* immunoglobulin G1, *TNF-α* tumor necrosis factor α, *CD* cluster of differentiation, *HER2* human epidermal growth factor receptor 2, *CDR* complementary-determining region, *c-Met* mesenchymal epithelial transition factor, it is also called tyrosine-protein kinase Met or hepatocyte growth factor receptor (HGFR), *CTLA-4* cytotoxic T-lymphocyte-associated protein 4, *VCAM-1* vascular cell adhesion molecule 1, *DM1* N2’-deacetyl-N2’-(3-mercapto-1-oxopropyl)-maytansine, *HIV* human immunodeficiency virus, *EGFR* epidermal growth factor receptor, *MW* molecular weight

### Special hindrance-based Ab lock

#### Autologous hinge domain

**Fig. 2:**
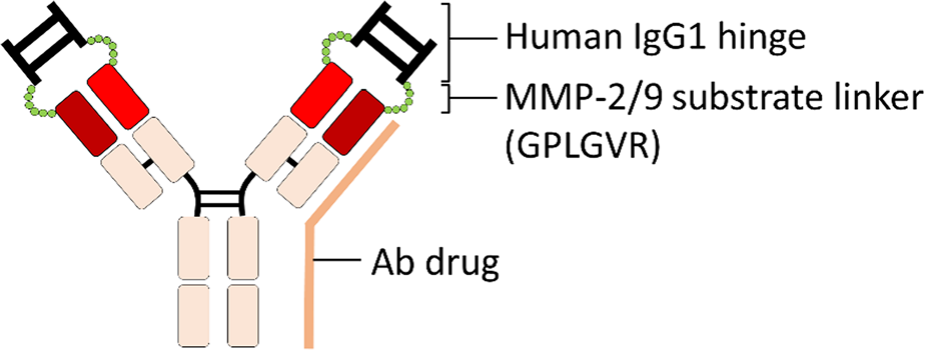
The autologous hinge domain as a universal Ab lock for enhancing disease site selectivity and minimizing the on-target toxicity of Ab drugs. Lu et al. used an autologous human IgG1 hinge domain as an Ab lock to cover the antigen binding site of a Ab drug (i.e., anti-TNF-α Ab, Infliximab) by using the MMP-2/9 substrate linker to generate a pro-Ab. When the pro-Ab encounters activated protease at the inflamed region (e.g., the RA region), the substrate linker is hydrolyzed, the Ab lock is released, and the pro-Ab can specifically activate and neutralize the target antigen (e.g., TNF-α) at the disease site to inhibit RA progression. Ab, antibody; IgG1, immunoglobulin G1; MMP, matrix metalloproteinase; TNF-α, tumor necrosis factor α

Lu and colleagues [100] used an autologous human immunoglobulin G1 (IgG1) hinge as a universal Ab lock to cover the TNF-α-binding site of Infliximab (anti-TNF-α Ab) by linking it with matrix metalloproteinase-2 and -9 (MMP-2/9) substrate (Gly-Pro-Leu-Gly-Val-Arg; GPLGVR) to generate Pro-Infliximab. Once the Pro-Infliximab encounters the overexpressed MMP-2/9 and is specifically hydrolyzed in the disease region of rheumatoid arthritis (RA), the cleaved Pro-Infliximab is specifically activated and neutralizes the target antigen to suppress RA progression (Fig. 2). The Ab lock significantly inhibited the TNF-α-binding ability of Pro-Infliximab by 395-fold as compared with the original Infliximab and MMP-2/9 can completely reactivate the TNF-α neutralizing ability of Pro-Infliximab to block TNF-α-induced nucleus factor kappa B (NF-κB) signaling [100]. Lu et al. also proved that Pro-Infliximab was only selectively and gradually activated at the disease site (i.e., mouse paws) but not other peripheral organs (e.g. peripheral blood, colon, lung or spleen) of a human TNF-α transgenic mouse model, which can spontaneously induce severe chronic arthritis at paws by approximately 20 weeks of age, and presented similar pharmacokinetics (PK) and bio-distribution to Infliximab in healthy DBA/1 mice [100]. Furthermore, Pro-Infliximab not only provided equivalent therapeutic efficacy to Infliximab, but also maintained mouse immunity against *Listeria* infection in a RA transgenic mouse model, leading to significantly higher survival rate (71%) than that of the Infliximab-treatment group (0%) [100]. In addition, Pro-Infliximab was able to significantly reduce the binding and neutralizing effect of anti-Infliximab idiotypic Ab, which is a major problem in Ab-based therapy after repeat administration of Ab drugs [21], by 108-fold as compared to original Infliximab, and the neutralizing activity of anti-Infliximab idiotypic Ab to Pro-Infliximab could be completely restored after MMP-2/9 cleavage, revealing that the autologous hinge domain forms a spatial barrier and protects antigen binding site of Ab drug from binding of corresponding anti-idiotypic Ab [100]. The spatial-hindrance-based Ab lock has the following advantages: (1) Can be widely applied to a variety of Ab drugs with different target antigens; (2) Autologous hinges may minimize the risk of immunogenicity of anti-Hinge Ab from the host; (3) Ab lock can significantly inhibit the binding and neutralizing ability of anti-idiotypic Ab to original Ab drugs, at least in the case of Infliximab; (4) The changeable design of protease substrate between Ab lock and Infliximab can be applied to any alternative Ab drugs that are used for RA or even other disease treatments. It is expected that a protease cleavable and efficient Ab lock can significantly increase the selective activation of Ab drugs at the disease site and reduce the on-target toxicities of Ab drugs during systemic circulation, thus potentially improving the clinical benefit and quality of life of patients.

Lu et al. used an autologous human IgG1 hinge domain as an Ab lock to cover the antigen binding site of a Ab drug (i.e., anti-TNF-α Ab, Infliximab) by using the MMP-2/9 substrate linker to generate a pro-Ab. When the pro-Ab encounters activated protease at the inflamed region (e.g., the RA region), the substrate linker is hydrolyzed, the Ab lock is released, and the pro-Ab can specifically activate and neutralize the target antigen (e.g., TNF-α) at the disease site to inhibit RA progression. Ab, antibody; IgG1, immunoglobulin G1; MMP, matrix metalloproteinase; TNF-α, tumor necrosis factor α.

#### Coiled-coil domain

**Fig. 3:**
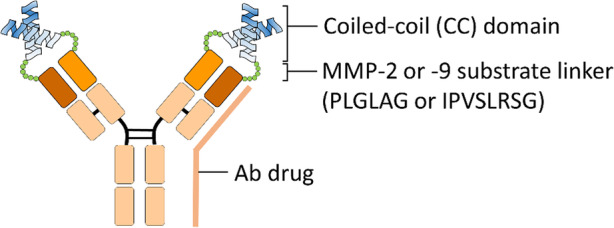
The coiled-coil domain as a universal Ab lock for enhancing the selectivity of Ab drugs to the disease site. Trang et al. used leucine-rich and parallel heterodimeric coiled-coil domains as an Ab lock that spatially occupies the antigen binding site of Ab drugs (i.e., anti-CD19 mAb, anti-CD20 mAb, anti-HER2 mAb, anti-αVβ6 mAb and anti-mouse CD3 mAb) by using MMP-2 or -9 substrate linker (PLGLAG or IPVSLRSG) to generate a pro-Ab. When protease activation occurs at the inflamed region (e.g., tumor region), the coiled-coil domain is released, and the pro-Ab can specifically revive and neutralize the target antigen (e.g., CD19, CD20, HER2, αVβ6 or CD3) at the disease site to inhibit disease progression. Ab, antibody; CD, cluster of differentiation; HER2, human epidermal growth factor receptor 2; MMP, matrix metalloproteinase

Trang and colleagues [176] also developed a generalizable approach for Ab masking with a leucine-rich and parallel heterodimeric coiled-coil domain that spatially occupies the antigen binding site of Ab drugs (Fig. 3). The coiled-coil (CC) masking domains with different inter-coil affinities or orientations were derived from de novo designs [173] or native human proteins [15, 149], such as c-Fos and c-Jun. In the initial test of this strategy, the authors evaluated the masking efficiency of parallel heterodimeric coils with low (CC1) and high (CC2A and 2B) inter-coil interaction [173], disulfide-linked coils (CC3) [149], antiparallel heterodimeric (CC4) [106] and helix-turn-helix homodimeric coils (CC5) [131] to a CD19-binding Ab hBU12 [53] by assessing the antigen binding ability to CD19-expressing Raji cells. The results suggested that parallel heterodimeric coiled-coils CC2A, CC2B and CC3 had excellent blocking activity of over 300-fold. In particular, CC2B was found more suitable for further developing Ab-drug conjugates (ADCs) because the lack of covalent disulfide linkages between the coiled-coil domains minimizes the heterogeneity of chemical drug attachment by using endogenous disulfide conjugation. Trang further linked CC2B peptide to all four N-terminals (two light chains and two heavy chains) of different Ab drugs, including Rituximab (anti-CD20 mAb) [80], Trastuzumab (anti-HER2 mAb) [105], h15H3 (anti-αVβ6 mAb) [176] and 145-2C11 (anti-mouse CD3 mAb) [88] with MMP-2 and MMP-9 cleavable sequence (PLGLAG [72] or IPVSLRSG [178]) and proved that the CC2B domain can significantly decrease the antigen binding affinity of each mAb at least 80-fold, 470-fold, 290-fold and 1000-fold, respectively. After treatment with purified MMP-2, the CC2B mask can be efficiently removed to restore the antigen binding ability of the Ab drugs to within 1.7-fold as compared to parent Ab. With ADCs, the incorporation of a protease-cleavable sequence between the coiled-coil mask and Ab drugs significantly improved the selectivity of Ab drugs to the disease site from normal healthy tissues, thereby increasing the pharmacokinetics and therapeutic efficacy by avoiding the effect of antigen sink in a xenograft tumor mouse model. Furthermore, the sterical-hindrance-based coiled-coil mask also has the following advantages: (1) Possible wide application to different Ab drugs in the clinic or in development; (2) Coiled-coil mask provides high masking efficiency to Ab drugs in a condition that lacks proteolytic enzymes and maintains the Ab level by avoiding the effect of antigen sink; (3) High release rate of the coiled-coil mask from Ab drugs after cleaving with disease-specific protease. However, the relatively complex helix structure and custom mutated coiled-coil mask may significantly reduce the productive rate and increase the risk of immunogenicity during long-term systemic administration of coiled-coil masked-Ab drugs.

Trang et al. used leucine-rich and parallel heterodimeric coiled-coil domains as an Ab lock that spatially occupies the antigen binding site of Ab drugs (i.e., anti-CD19 mAb, anti-CD20 mAb, anti-HER2 mAb, anti-αVβ6 mAb and anti-mouse CD3 mAb) by using MMP-2 or − 9 substrate linker (PLGLAG or IPVSLRSG) to generate a pro-Ab. When protease activation occurs at the inflamed region (e.g., tumor region), the coiled-coil domain is released, and the pro-Ab can specifically revive and neutralize the target antigen (e.g., CD19, CD20, HER2, αVβ6 or CD3) at the disease site to inhibit disease progression. Ab, antibody; CD, cluster of differentiation; HER2, human epidermal growth factor receptor 2; MMP, matrix metalloproteinase.

#### Non-antibody protein fragment

**Fig. 4:**
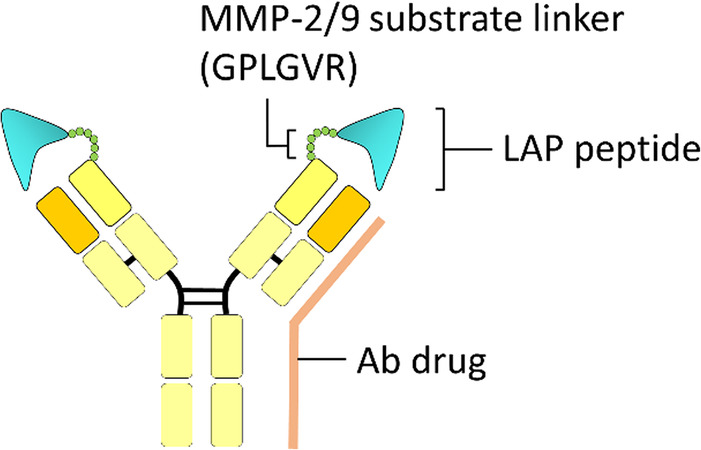
Endogenous LAP peptide is a widely applied Ab lock for enhancing the selectivity of Ab drugs to the disease site. Chen et al. used latency-associated peptide (LAP) derived from transforming growth factor-β (TGF-β) as a widely applied Ab lock and connected it to the N-terminal of the heavy chain of Ab drugs (e.g., the anti-EGFR Ab, Cetuximab, or the anti-TNF-α Ab, Infliximab) by a substrate peptide for MMP-2 (i.e., GPLGVR). The LAP domain reduced the antigen binding activity of Ab drugs and was selectively activated by MMP-2 for specific antigen targeting at the disease site. Ab, antibody; EGFR, epidermal growth factor receptor; TNF-α, tumor necrosis factor α; MMP, matrix metalloproteinase

Chen and colleagues [27] developed protease-activated pro-Ab by masking the antigen binding site of anti-EGFR Ab (Cetuximab) or anti-TNF-α Ab (Infliximab), respectively, with latency-associated peptide (LAP) derived from transforming growth factor-β (TGF-β) and connected it to the N-terminal of heavy chain of Ab by a substrate peptide for MMP-2 (i.e., GPLGVR) (Fig. 4). The LAP domain reduced the antigen binding activity of anti-EGFR Ab and anti-TNF-α Ab by 53.8 and 53.9%, respectively. After MMP-2 digestion of LAP-masked anti-EGFR Ab or LAP-masked anti-TNF-α Ab, the antigen binding ability rose progressively to levels similar to that of the unmodified Ab. The advantages of this masking strategy are that (1) the LAP domain derived from endogenous TGF-β proteins may reduce the opportunity of immunogenicity from the host; (2) the inhibitory domain displays no apparent or known biological function other than blocking the activity of the original proteins and can minimize unpredictable adverse events or cross reaction to other proteins. However, the poor masking efficiency of the LAP domain (only masks 1.86-fold) to the EGFR, or TNF-α binding ability of the original Ab drugs, and the reduced productive yield (retained only 33% productive yield as compared with original Ab) affected by significantly increased molecular weight (MW) of LAP-masked Ab (approximately 240 kDa) may increase difficulty in obtaining enough pro-Abs to investigate the PK, bio-distribution, therapeutic efficacy and on-target toxicities in living disease models and limit its broad application to other Ab drugs.

Chen et al. used latency-associated peptide (LAP) derived from transforming growth factor-β (TGF-β) as a widely applied Ab lock and connected it to the N-terminal of the heavy chain of Ab drugs (e.g., the anti-EGFR Ab, Cetuximab, or the anti-TNF-α Ab, Infliximab) by a substrate peptide for MMP-2 (i.e., GPLGVR). The LAP domain reduced the antigen binding activity of Ab drugs and was selectively activated by MMP-2 for specific antigen targeting at the disease site. Ab, antibody; EGFR, epidermal growth factor receptor; TNF-α, tumor necrosis factor α; MMP, matrix metalloproteinase.

#### Ab fragment

**Fig. 5:**
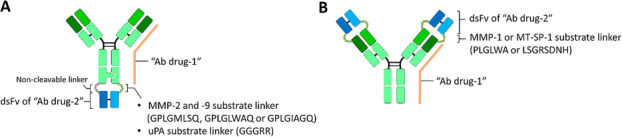
Ab fragment as an Ab lock for shielding antigen binding ability of specific Ab drugs. **a** Metz et al. constructed a trivalent bi-specific Ab by linking a disulfide-stabilized variable fragment (dsFv), which is specific against one antigen (e.g., c-Met), to the C-terminus of the heavy chain of a whole Ab (e.g., anti-HER3 Ab) with a one-armed protease substrate peptide (e.g., GPLGMLSQ, GPLGLWAQ and GPLGIAGQ for MMP-2 and MMP-9 or GGGRR for urokinase plasminogen activator (uPA)). In this design, the antigen binding ability of dsFv was sterically interfered with by the Fc portion of the front whole Ab until protease cleavage and reactivation of the dsFv activity. **b** Onuoha’s and Pai’s groups developed an activatable dual variable domain (aDVD) Ab by linking the dsFv from one Ab (e.g., anti-ICAM-1 Ab or anti-PSCA Ab) to the N-terminus of light and heavy chains of another Ab (e.g., anti-TNF-α Ab or anti-CTLA-4 Ab) with MMP-1- (PLGLWA) or MT-SP1-cleavable sequence (LSGRSDNH). The outer dsFv can significantly shield the antigen binding ability of the inner Ab and lead the aDVD to the disease site (e.g., tumor site), to be reactivated by disease-specific proteases and restore the antigen neutralizing ability of inner Ab drugs. Ab, antibody; HER3, human epidermal growth factor receptor 3; TNF-α, tumor necrosis factor α; MMP, matrix metalloproteinase; MT-SP1, activated membrane type-serine protease 1; ICAM-1, intercellular adhesion molecule 1; CTLA-4, cytotoxic T-lymphocyte-antigen 4

Metz et al. [108] engineered a trivalent bi-specific Ab by linking a disulfide-stabilized variable fragment (dsFv), which is specific against c-Met antigen that is deregulated in several types of cancer [110], to the C-terminus of the heavy chain of a bivalent anti-HER3 Ab with one-armed protease substrate peptide (GPLGMLSQ, GPLGLWAQ and GPLGIAGQ for MMP-2 and MMP-9 [118], and GGGRR for urokinase plasminogen activator (uPA) [28]). In this design, the c-Met binding ability of dsFv was sterically interfered with by the Fc portion of the front anti-HER3 Ab. After proteolytic processing by the corresponding protease, the dsFv is swiveled open and recovers its c-Met binding activity (Fig. 5a). The authors were able to illustrate an approximate 1000-fold difference in affinity between the pro-Ab format and the protease-activated form in vitro, suggesting that this trivalent bi-specific Ab may enhance tumor targeting as a result of hydrolyzation by proteases overexpressed in tumor microenvironment.

Similar to the strategy of activatable trivalent bi-specific Ab, Onuoha and colleagues [124] developed an activatable dual variable domain (aDVD) Ab by linking the dsFv from Ab against intercellular adhesion molecule 1 (ICAM-1) to the N-terminus of light and heavy chain of anti-TNF-α Ab (Infliximab and Adalimumab) with MMP-1-cleavable sequence (PLGLWA) (Fig. 5b). In this strategy, the outer arm of the anti-ICAM-1 variable domain retained its antigen binding ability and significantly masked the TNF-α binding ability of Infliximab or Adalimumab in the inner arm by greater than 1000-fold in K_D_ as measured by surface plasmon resonance (SPR) in vitro. The authors further proved that the human synovial fluid and physiologic concentrations of MMP-1 enzyme sufficiently cleaved and restored the TNF-α binding ability of aDVD and locally accumulated aDVD in the disease region. Pai et al. [125] also applied the aDVD strategy to anti-CTLA-4 Ab, which is an immune checkpoint inhibitor that specifically neutralizes CTLA-4 on activated T cells, enhancing the anti-cancer immunity, and forming anti-CTLA-4 aDVD. The CTLA-4 binding domain of the inner anti-CTLA-4 Ab is shielded by an outer dsFv specifically targeting the prostate stem cell antigen (PSCA) and linking with an activated membrane type-serine protease 1 (MT-SP1)-cleavable sequence (LSGRSDNH) (Fig. 5b). The outer anti-PSCA dsFv can significantly shield the CTLA-4 binding ability of the inner Ab and selectively increase CTLA-4-neutralizing activity by 25-fold as compared to the intact aDVD form after MT-SP-1 cleavage. The anti-CTLA-4 aDVD exhibits a potent anti-cancer effect by increasing tumor-infiltrating CD8^+^ T cells and decreasing the tumor-infiltrating Treg population in allogenic tumor-bearing mice and further preventing treatment-induced multiorgan toxicity. As with the activatable bi-specific Ab, the substrate linker of aDVD can also be specifically hydrolyzed by overexpressed protease in the inflamed region, restore the antigen neutralizing ability (e.g., TNF-α or CTLA-4) and potentially enhance the therapeutic index of Ab drugs for the treatment of RA, malignancies and other inflammatory diseases in the future. However, the antigen binding ability of the outer domain of trivalent bi-specific Ab or aDVD may still contribute to undesirable targeting of antigens by the masked Ab to non-diseased tissue and further induce unpredictable adverse events.

(A) Metz et al. constructed a trivalent bi-specific Ab by linking a disulfide-stabilized variable fragment (dsFv), which is specific against one antigen (e.g., c-Met), to the C-terminus of the heavy chain of a whole Ab (e.g., anti-HER3 Ab) with a one-armed protease substrate peptide (e.g., GPLGMLSQ, GPLGLWAQ and GPLGIAGQ for MMP-2 and MMP-9 or GGGRR for urokinase plasminogen activator (uPA)). In this design, the antigen binding ability of dsFv was sterically interfered with by the Fc portion of the front whole Ab until protease cleavage and reactivation of the dsFv activity. (B) Onuoha’s and Pai’s groups developed an activatable dual variable domain (aDVD) Ab by linking the dsFv from one Ab (e.g., anti-ICAM-1 Ab or anti-PSCA Ab) to the N-terminus of light and heavy chains of another Ab (e.g., anti-TNF-α Ab or anti-CTLA-4 Ab) with MMP-1- (PLGLWA) or MT-SP1-cleavable sequence (LSGRSDNH). The outer dsFv can significantly shield the antigen binding ability of the inner Ab and lead the aDVD to the disease site (e.g., tumor site), to be reactivated by disease-specific proteases and restore the antigen neutralizing ability of inner Ab drugs. Ab, antibody; HER3, human epidermal growth factor receptor 3; TNF-α, tumor necrosis factor α; MMP, matrix metalloproteinase; MT-SP1, activated membrane type-serine protease 1; ICAM-1, intercellular adhesion molecule 1; CTLA-4, cytotoxic T-lymphocyte-antigen 4.

### Affinity-peptide based Ab lock

#### Affinity peptide

**Fig. 6:**
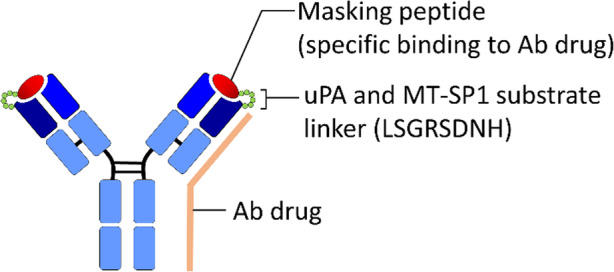
Affinity peptide-based Ab lock for masking the antigen binding activity of Ab drugs. CytomX therapeutics, Inc. developed PROBODY therapeutics by masking the antigen binding site of Ab drugs (e.g., anti-EGFR Ab, anti-VCAM-1 Ab or anti-EGFR ADC) with an affinity peptide screened out from phage display library technology and linked it to an Ab through a protease substrate peptide (e.g., LSGRSDNH for uPA and MT-SP1). The Pro-Ab exhibits weaker antigen binding ability than the original Ab drug, maintains intact format in systemic circulation and the biological activity could be specifically restored in the tumor microenvironment in a protease dependent manner. Ab, antibody; EGFR, epidermal growth factor receptor; ADC, antibody-drug conjugate; VCAM-1, vascular cell adhesion molecule 1; uPA, urokinase plasminogen activator; MT-SP1, activated membrane type-serine protease 1

The most rapidly progressing affinity-based pro-Ab strategy is PROBODY therapeutics developed by CytomX therapeutics, Inc. The target binding region of the mAb is masked by an affinity peptide screened out using phage display library technology and linked through a protease substrate peptide which allows it be cleaved in disease microenvironment (Fig. 6). The Probody therapeutic platform ensures the Ab drugs can only be activated in diseased tissue but not in normal healthy tissues, thereby preventing unpredictable side effects caused by systemic reactions in traditional Ab drugs. For example, Desnoyers and colleagues generated an epithelial growth factor receptor (EGFR) pro-Ab by selecting a binding peptide from a bacterial peptide display library that could specifically mask the antigen-binding site of anti-EGFR Ab (Cetuximab, Erbitux®) and linking with substrate peptide of tumor-associated protease, urokinase-type plasminogen activator (uPA) [35]. The EGFR pro-Ab exhibits 48-fold weaker antigen binding ability than the original Cetuximab, maintains an intact format in systemic circulation and the biological activity could be specifically restored in the tumor microenvironment in a protease dependent manner. The authors also proved that the EGFR Probody therapeutic can increase drug exposure in xenograft tumors by avoiding target-mediated drug disposition (TMDD) in mouse models, reduce the skin toxicity associated with parental Cetuximab and elevate the safety index 3- to 15-fold during Probody treatment in cynomolgus monkeys. Similar strategies were also applied to other Abs such as anti-vascular cell adhesion molecule 1 (VCAM-1) mAb [43] and anti-EGFR mAb (Panitumumab, Vectibix®) [193]. Furthermore, Liu et al. [93] and Yang et al. [192] applied the Probody approach to the maytansine (DM1)-conjugated anti-EGFR mAb (Panitumumab) and generated a pro-antibody-drug conjugate (PDC) named PanP-DM1. Regardless of DM1 conjugation, the PanP-DM1 still displayed 12-fold weaker binding ability to immobilized EGFR antigen than parental Panitumumab and significantly improved the cancer-selective activity, therapeutic and safety index over traditional ADC. Collectively, the mouse and cynomolgus monkey data indicate that the Probody therapeutics can significantly expand the therapeutic window of mAb therapy. However, these exogenous and affinity-based masking peptides may not be efficiently released from pro-antibodies after protease cleavage due to the binding ability of masking peptides to antibodies, and the exogenous property may also increase the risk of undesirable immune responses from the host. In addition, the customized masking peptide may limit the Probody approach from being widely applied to different Ab drugs in the development stage or in the clinic.

CytomX therapeutics, Inc. developed PROBODY therapeutics by masking the antigen binding site of Ab drugs (e.g., anti-EGFR Ab, anti-VCAM-1 Ab or anti-EGFR ADC) with an affinity peptide screened out from phage display library technology and linked it to an Ab through a protease substrate peptide (e.g., LSGRSDNH for uPA and MT-SP1). The Pro-Ab exhibits weaker antigen binding ability than the original Ab drug, maintains intact format in systemic circulation and the biological activity could be specifically restored in the tumor microenvironment in a protease dependent manner. Ab, antibody; EGFR, epidermal growth factor receptor; ADC, antibody-drug conjugate; VCAM-1, vascular cell adhesion molecule 1; uPA, urokinase plasminogen activator; MT-SP1, activated membrane type-serine protease 1.

#### Cross-masking antibodies

**Fig. 7:**
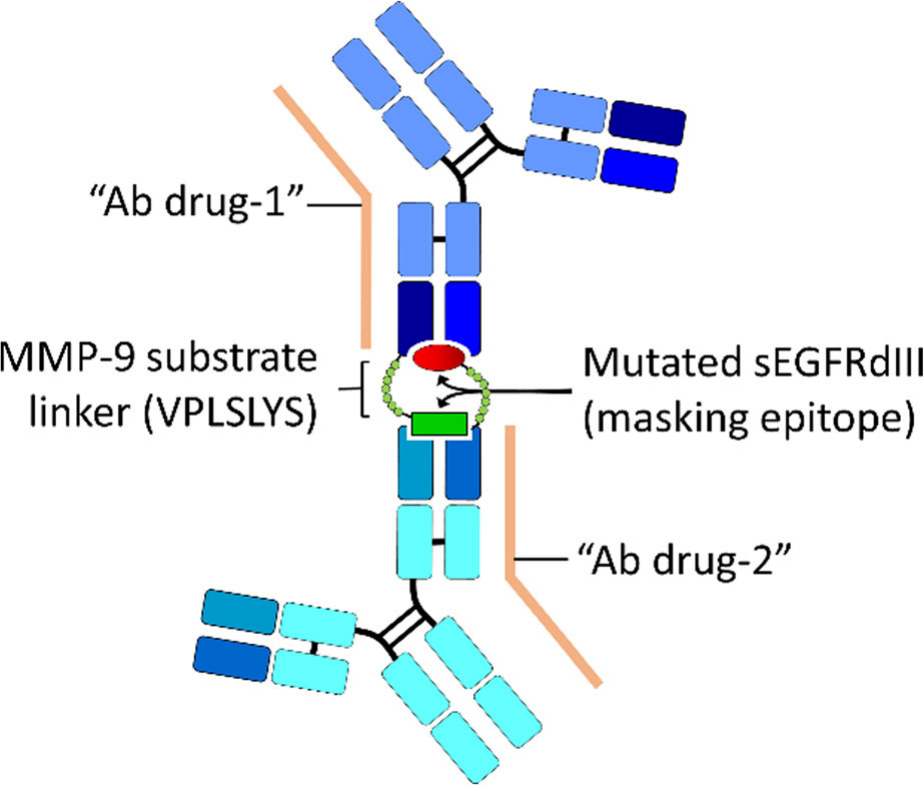
Cross-masking antibodies for limiting off-target effects of Ab drugs. Donaldson et al. used a cross-masking Ab approach to simultaneously block two Ab drugs. In this design, one Ab drug was linked with a corresponding antigen epitope (e.g., mutated soluble EGFR domain III (sEGFRdIII)) of another Ab drug via a protease-specific substrate peptide (VPLSLYS for MMP-9) and vice versa. The mutated masking epitope can significantly reduce the binding affinity of the linked Ab drug to a specific antigen and increase the releasing efficiency from the cross-masking Ab after MMP-9 cleavage, thereby restoring the antigen-neutralizing activity of the Ab drug. Ab, antibody; EGFR, epidermal growth factor receptor; MMP, matrix metalloproteinase

The cross-masking Ab approach involves mutual masking of two different Ab drugs. In this design, one Ab drug is linked with a corresponding antigen epitope of another Ab drug via protease-specific substrate peptide and vice versa (Fig. 7). Donaldson and colleagues [36] conducted a proof-of-concept study for this strategy in vitro by using two single chain Fv (scFv) derived from two anti-EGFR Abs (Cetuximab and Matuzumab) and linking with a point mutated soluble EGFR domain III (sEGFRdIII) (S460P/G461N for masking Cetuximab and Q384A/Q408M/H409E for masking Matuzumab) through MMP-9 substrate peptide, respectively. The mutation of sEGFRdIII significantly reduced the binding affinity of linked anti-EGFR scFv from 420 ± 270 nM to 3500 ± 1.1 nM and increased the releasing efficiency from cross-masking Ab after MMP-9 cleavage. After purifying and mixing together individual constructs, it allowed the assembly of the cross-masking Ab complex. The SPR and Flow cytometry data suggested that the cross-masking Abs poorly interacted (approximately 8.3-fold weaker than parental scFv) with native EGFR antigen and the binding ability was restored after MMP-9 treatment as compared with the original anti-EGFR scFv. The cross-masking strategy permits the simultaneous delivery of two Ab drugs that synergize or independently target two different tumor-associated antigens and may increase the therapeutic efficiency of diseases. However, the low masking efficiency, structural complexity, and homogeneity of cross-masking Ab formation and customized mutation and unpredictable releasing rate of the masking domain may limit its wide application to different Ab drugs.

Donaldson et al. used a cross-masking Ab approach to simultaneously block two Ab drugs. In this design, one Ab drug was linked with a corresponding antigen epitope (e.g., mutated soluble EGFR domain III (sEGFRdIII)) of another Ab drug via a protease-specific substrate peptide (VPLSLYS for MMP-9) and vice versa. The mutated masking epitope can significantly reduce the binding affinity of the linked Ab drug to a specific antigen and increase the releasing efficiency from the cross-masking Ab after MMP-9 cleavage, thereby restoring the antigen-neutralizing activity of the Ab drug. Ab, antibody; EGFR, epidermal growth factor receptor; MMP, matrix metalloproteinase.

#### Bivalent peptide-double strand DNA (dsDNA) conjugates

**Fig. 8:**
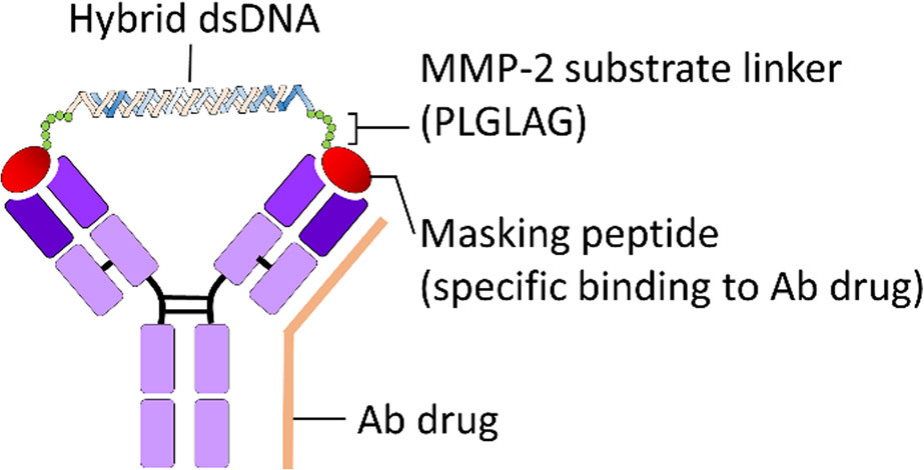
Bivalent peptide-double strand DNA (dsDNA) conjugates as affinity-based Ab lock for blocking antigen binding ability of Ab drugs. Janssen et al. generated a protease-activatable bivalent peptide-double strand DNA (dsDNA) conjugate as a masking molecule of Ab for reversible blocking effect on antigen binding ability of specific Ab drugs. In this design, the masking molecule contains two binding epitopes of a specific Ab drug (e.g., anti-HIV p17 mAb) and conjugates to a 35 base-paired single strand DNA (ssDNA) by linking with a maleimide functionalized MMP-2-cleavable sequence (e.g., maleimide-PLGLAG). The two complementary ssDNA fragments can naturally hybridize to form double helix and effectively bridge the masking epitope between two antigen binding sites within the same Ab, forming an Ab-ligand complex. After MMP-2 cleavage in the disease region, the masking molecule is released and the Pro-Ab is also reactivated and performs its original therapeutic function. mAb, monoclonal antibody; HIV, human immunodeficiency virus; MMP, matrix metalloprotease

Janssen and colleagues generated a protease-activatable bivalent peptide-double strand DNA (dsDNA) conjugate as an Ab masking molecule to achieve a reversible blocking effect on antigen binding ability of specific Ab drugs [70]. In this design, the masking molecule contains two binding epitopes (ELDRWEKIRLRP) of anti-HIV p17 mAb and conjugates to a 35 base-paired single strand DNA (ssDNA) by linking with a maleimide functionalized MMP-2-cleavable sequence (maleimide-PLGLAG). The two complementary ssDNA fragments can naturally hybrid to form a double helix and effectively bridge the masking epitope between two antigen-binding sites within the same Ab, forming an Ab-ligand complex (Fig. 8). Janssen proved that the bivalent peptide-dsDNA conjugates can efficiently mask the p17 binding affinity by 8-fold of the model anti-HIV p17 mAb as compared to parental Ab, and the blockage effect of bivalent peptide-dsDNA conjugates was nearly completely restored after cleaving with MMP-2 within 2 h in an in vitro binding assay. However, the low masking efficiency, heterogeneity of Ab-ligand complex formation after mixing constant ratio of bivalent masking molecule and Ab drugs, customized masking epitope, immunogenicity and uncertainty releasing rate of masking peptide may restrict its broad application to various Ab drugs.

Janssen et al. generated a protease-activatable bivalent peptide-double strand DNA (dsDNA) conjugate as a masking molecule of Ab for reversible blocking effect on antigen binding ability of specific Ab drugs. In this design, the masking molecule contains two binding epitopes of a specific Ab drug (e.g., anti-HIV p17 mAb) and conjugates to a 35 base-paired single strand DNA (ssDNA) by linking with a maleimide functionalized MMP-2-cleavable sequence (e.g., maleimide-PLGLAG). The two complementary ssDNA fragments can naturally hybridize to form double helix and effectively bridge the masking epitope between two antigen binding sites within the same Ab, forming an Ab-ligand complex. After MMP-2 cleavage in the disease region, the masking molecule is released and the Pro-Ab is also reactivated and performs its original therapeutic function. mAb, monoclonal antibody; HIV, human immunodeficiency virus; MMP, matrix metalloprotease.

### Comparison of different masking strategies of Pro-Abs

Development of approaches to address on-target toxicity caused by systemic neutralization of antigens by Ab drugs has generated a new vision in Ab drug development. The generation of Pro-Abs by installing a protease-cleavable Ab lock is a novel and advanced recombinant Ab-based strategy for selective activation of Ab drugs at the disease site. For good design of an Ab lock, multiple factors should be considered, including the masking efficiency, immunogenicity, wide applicability, Ab lock release efficiency after protease cleavage and reduction of side effects (Table 3). Spatial-hindrance-based Ab locks, such as autologous hinge domain, coiled-coil domain, LAP domain or trivalent/tetravalent Ab-based approaches, provide high masking efficiency, high release rate of masking domain and high restoration of antigen binding activity of mAbs after protease cleavage and nearly universal application of various Ab drugs. Affinity-based masking strategies (i.e., masking dependent on affinity peptides or mutant antigens) demonstrate acceptable masking efficiency; however, custom design of masking peptides used in individual mAbs and uncertain release rate of the masking molecule after protease hydrolysis may make it impractical for clinical applications. Depending on the source of the masking domain of Pro-Abs, the Ab lock is involved in the intracellular (i.e., c-Fos and c-Jun) or artificial coiled-coil domain, exogenous Ab fragment in trivalent/tetravalent Ab-based approaches, masking peptide derived from phage display and mutated antigen may increase the risk of immunogenicity during Pro-Ab treatment. In principle, both masking theories (i.e., spatial-hindrance-based or affinity peptide-based approaches) can inhibit the neutralizing and cleaning effect of anti-idiotypic anti-drug Abs, which is one of major problems in Ab therapy, caused by repeated administration of mAb. Lu’s team provided evidence that an autologous hinge can efficiently prevent pro-Infliximab from the neutralizing activity of anti-idiotypic Ab and the TNF-α binding ability can be completely restored by MMP-2/9 cleavage. Most importantly, research groups that developed autologous hinge domain-based, Ab fragment in trivalent/tetravalent Ab-based and phage display-derived masking peptide-based approaches provide evidence that these three Ab lock can efficiently mask antigen binding ability of Ab drugs, and prevent systemic antigen targeting, thereby significantly reducing the side effects caused by the mechanism-of-action of Ab drugs. In summary, there are different advantages with different masking strategies of Pro-Ab; they may provide scientists more options for developing next generation Ab drugs corresponding to the conditions of different type of diseases.

**Table 3:** Comparison of different masking strategies of pro-Abs

Masking concept	Spatial hindrance-based Ab lock				Affinity peptide-based Ab lock	
Ab lock	Autologous hinge domain	Coiled-coil (CC) domain	Non-Ab protein fragment (e.g., LAP)	Ab fragment (e.g., dsFv)	Affinity peptide	Mutated antigen
Schematic						
MW of Ab lock	3.5 kDa	5 ~ 9.2 kDa	40 kDa	26 kDa	2.8 ~ 5 kDa	23.3 kDa
Masking efficiency (compared with parental Ab)	> 390 fold	80 ~ 1000 fold	> 1.9 fold	25 ~ 1000 fold	8 ~ 48 fold	8.3 fold
Immunogenicity	Low	Risk	Low	Risk	Risk	Risk
Wide applicability	Yes	Yes	Yes	Yes	Customized	Customized
Ab lock released efficiency (after protease cleaved)	Good	Good	Good	Good	Concerned	Concerned
Evidence for preventing anti-idiotypic Ab binding	Yes	n.d.a.	n.d.a.	n.d.a.	n.d.a.	n.d.a.
Evidence for reducing side effect in vivo	Yes	n.d.a.	n.d.a.	Yes	Yes	n.d.a.

*LAP* latency-associated peptide, *dsFv* disulfide-stabilized variable fragment, *H* heavy chain, *L* light chain, *MW* molecular weight, *n.d.a.* No data available

### Disease-specific proteases locally unlock and reactivate pro-antibodies in the disease region

Proteases are an attractive target for drug development and deregulation of protease activity has been involved in the pathogenesis of a variety of diseases including malignancies, autoimmune diseases and inflammatory disorders (Table 4). In the field of oncology, for example, the expression of MMP-2 and MMP-9, which are members of the endopeptidase family that can degrade different components of the extracellular matrix (ECM) [87], are upregulated by acute lymphoblastic leukemia (ALL) cells and hydrolyze the basement membrane of blood vessels, releasing matrix-bound vascular endothelial growth factor (VEGF), inducing angiogenesis and stimulating extramedullary infiltration by lymphoblasts [133]. Similarly, MMP-9 plays an important role in migration and survival, and speeds up the disease progression of chronic lymphocytic leukemias (CLL) [11]. MMP-2, MMP-9 and MMP-13 are also reported to be overexpressed in breast cancer patients and correlated with more aggressive phenotypes of breast cancer, poor prognosis and decreased life expectancy [49, 138, 141]. Cathepsin B has been reported to be highly expressed in breast cancer patients and mainly responsible for degrading collagen, fibronectin, laminin and proteoglycans, thereby promoting nodal metastasis [48, 120, 158]. The urokinase plasminogen activator (uPA) system, which represents a family of serine protease, also participates to tumorigenesis. uPA has been reported to be overexpressed in several type of cancers, such as esophageal [156], gastric [71], colorectal [159], pancreatic [119], breast [40, 96, 162], cervical [82], ovarian [90], prostate [134], leukemia [148], brain [64] and renal cancers [168]. It can degrade the basement membrane or ECM around the tissue by itself or further activate the downstream MMP family [33]. The higher uPA level in cancer patients increases the frequency of metastasis and leads to poor prognosis and survival rate [33]. MMPs also serve as inflammatory markers that are involved in the pathogenesis of several autoimmune disorders or chronic conditions. For instance, the expression level of MMP-2 and MMP-9 are increased in the synovial liquid of patients with RA and contribute to the destruction of the cartilage, tendons, and bone in the synovial joints [9, 16, 164, 194]. MMP-1, MMP-8, MMP-9 and MMP-12 have been shown to be upregulated in the disease region of chronic obstructed pulmonary disease (COPD), which is a chronic inflammatory airway disorder commonly caused by cigarette smoking and occupational inhalation (e.g., dust or chemicals) [139]. The overexpressed MMP may increase the ECM degradation in the lungs and therefore accelerate the loss of lung function [142, 155]. It is notable that deregulation of protease activity is an important hallmark of most diseases because it may be required for tissue repair or disease progression [1, 3, 5, 7, 10, 12, 14, 17, 25, 26, 31, 32, 38, 39, 45, 46, 50, 52, 56–58, 62, 68, 75, 76, 83, 84, 86, 89, 92, 104, 107, 109, 112, 117, 121–123, 126, 127, 136, 144, 150, 151, 153, 157, 160, 163, 166, 167, 174, 177, 184, 189, 190, 196–198]. Based on the changeable design of the pro-Ab therapeutic strategy, it can be widely applied to different kinds of diseases by replacing the substrate linker on pro-Ab to correspond to the substrate sequence of specific proteases, which are overexpressed in the disease of interest.

**Table 4:** Published disease specific “Un-lock” proteases

Disease	Protease expression (Refs.)	Example of applicable Ab drugs
**Oncology**		
Lung cancer	MMP-2 [17], MMP-13 [144], MMP-14 [49], Cathepsin B [76, 163, 184], Cathepsin H [151], Cathepsin S [83], ADAM-8 [68], ADAM-9 [117], ADAM-15 [150], ADAM-17 [117], ADAM-28 [122]	• Nivolumab/Pembrolizumab (anti-PD-1 Ab) • Atezolizumab/Durvalumab (anti-PD-L1 Ab) • Necitumumab (anti-EGFR Ab) • Bevacizumab (anti-VEGF-A Ab) • Ramucirumab (anti-VEGFR2 Ab)
Colorectal cancer	MMP-2 [49], MMP-7 [26], MMP-9 [52, 197], MMP-13 [189], Cathepsin B [1, 177], Cathepsin L [1, 177], Cathepsin S [57], ADAM-10 [50], ADAM-12 [117], ADAM-17 [12], uPA [159]	• Cetuximab/Panitumumab (anti-EGFR Ab) • Bevacizumab (anti-VEGF-A Ab) • Ramucirumab (anti-VEGFR2 Ab) • Ipilimumab (anti-CTLA-4 Ab) • Nivolumab/Pembrolizumab (anti-PD-1 Ab)
Breast Cancer	MMP-2 [49, 138], MMP-9 [49, 138], MMP-13 [141], MMP-14 [49], Cathepsin B [46, 48, 62, 84, 120, 158, 174], Cathepsin C [123], Cathepsin L [46, 126, 174], Cathepsin S [153], ADAM-9 [121], ADAM-12 [117], ADAM-15 [86], ADAM-17 [107], ADAM-28 [112], uPA [40, 96, 162]	• Trastuzumab/Trastuzumab emtansine (T-DM1)/Pertuzumab (anti-HER2 Ab) • Atezolizumab (anti-PD-L1 Ab)
**Autoimmune disorders**		
Rheumatoid arthritis (RA)	MMP-2 [16], MMP-3 [9, 164, 194], MMP-9 [16],	• Adalimumab/Infliximab/Golimumab/Certolizumab pegol (anti-TNF-α Ab) • Sarilumab/Tocilizumab (anti-IL-6R Ab) • Rituximab (anti-CD20 Ab) • Secukinumab (anti-IL-17A Ab)
Psoriasis	MMP-1 [160], MMP-2 [45, 157, 160], MMP-3 [25], MMP-7 [109], MMP-8 [38], MMP-9 [5, 14, 45, 157, 160], MMP-12 [160, 167], MMP-14 [45], MMP-15 [45], MMP-19 [166], MMP-26 [167], MMP-28 [196], ADAM-10 [109], ADAM-12 [109], ADAM-17 [75], ADAM-33 [3, 198]	• Adalimumab/Infliximab (anti-TNF-α Ab) • Efalizumab (anti-CD11a Ab) • Ustekinumab (anti-IL-12/23 Ab) • Secukinumab/Ixekizumab (anti-IL-17A Ab) • Brodalumab (anti-IL-17R Ab) • Guselkumab/Tildrakizumab/Risankizumab (anti-IL-23 p19 Ab)
Multiple sclerosis (MS)	MMP-2 [7], MMP-7 [31, 92], MMP-9 [7, 31, 32, 56, 58, 89, 92]	• Natalizumab (anti-α4 integrin Ab) • Alemtuzumab (anti-CD52 Ab) • Ocrelizumab (anti-CD20 Ab) • Daclizumab (anti-CD25 Ab)
**Inflammatory diseases**		
Chronic obstructed pulmonary disease (COPD)	MMP-1 [139], MMP-7 [155], MMP-8 [139], MMP-9 [139, 142, 155], MMP-12 [139]	• Mepolizumab (anti-IL-5 Ab) • Canakinumab (anti-IL-1β Ab) (Phase 1 clinical trial)
Inflammatory bowel disease (IBD)	MMP-1 [127], MMP-3 [127], MMP-7 [127, 136], MMP-9 [104, 127], MMP-10 [127], MMP-12 [127], MMP-13 [136]	• Adalimumab/Infliximab/ Golimumab/Certolizumab pegol (anti-TNF-α Ab) • Vedolizumab (anti-α4β7 integrin Ab) • Natalizumab (anti-α4 integrin Ab) • Ustekinumab (anti-IL-12/23 Ab)
**Chronic diseases**		
Osteoporosis	MMP-1 [10], MMP-2 [10], MMP-9 [10], MMP-13 [10], MMP-14 [10], Cathepsin K [39]	• Denosumab (anti-RANKL Ab) • Romosozumab (anti-Sclerostin Ab)
Alzheimer’s disease (AD)	BACE1 [190]	• Aducanumab (anti-Aβ Ab) (Phase 3 clinical trial)

*MMP* matrix metalloprotease, *ADAM* a disintegrin and metalloproteinase, *uPA* urokinase plasminogen activator, *PD-1* programmed cell death protein 1, *PD-L1* programmed death-ligand 1, *EGFR* epidermal growth factor receptor, *VEGF-A* vascular endothelial growth factor A, *VEGFR2* vascular endothelial growth factor receptor 2, *CTLA-4* cytotoxic T-lymphocyte-associated protein 4, *HER2* human epidermal growth factor receptor 2, *TNF-α* tumor necrosis factor α, *IL* interleukin, *CD* cluster of differentiation, *IL-6R* IL-6 receptor, *RANKL* receptor activator of nuclear factor kappa-Β ligand, *BACE1* β-site amyloid β precursor protein cleaving enzyme 1, *Aβ* Amyloid β

### The pro-antibody strategy avoids the inhibitory effect of anti-idiotypic anti-drug antibodies

A major limitation of mAb therapy is generation of anti-idiotypic Abs after repeated administration of Ab drugs. Despite the development of humanized and fully human mAbs that try to minimize immunogenic activity, human anti-human idiotypic Abs (also known as anti-idiotypic Abs) are still generated against the idiotypic domain on the antigen binding site of mAbs. For example, about 12% of RA patients received Adalimumab (a fully human IgG1 mAb against human TNF-α) treatment that tested positive for ADA against Adalimumab [113, 179]. This kind of ADA may result in the formation of immune complexes, which accelerate its clearance from circulation, thereby reducing the drug’s half-life or directly blocking the binding ability of Adalimumab to its target, reducing treatment efficacy. Van Schouwenburg and colleagues even suggest that at least 98% of anti-Adalimumab Abs are capable of neutralizing this agent [180]. Davda et al. indicated that the low incidence of ADA (0–12.7%), which contains 0–0.8% neutralizing ADA, was found following a single-agent treatment with immune checkpoint inhibitors such as anti-PD-1 mAbs (Nivolumab, Pembrolizumab, and Cemiplimab); the anti-CTLA-4 mAb (Ipilimumab); and the anti-PD-L1 mAbs (Avelumab and Durvalumab) in patients with advanced malignancies [2, 34]. However, a high incidence of ADA (23.8–37.8%), which contains 0.5–4.6% neutralizing ADA, was observed against Nivolumab when combining an anti-PD-1/PD-L1 and an anti-CTLA-4 mAb in patients with advanced solid tumors [34]. Fortunately, there were no clinically relevant effects on the safety, pharmacokinetics, or therapeutic efficacy of Nivolumab in patients who developed anti-Nivolumab Abs [34]. It should be noted that the immunogenicity of mAbs is not a simple issue about the sequence homology to human Abs. In the study from Lu et al., the spatial-hindrance-based Ab lock (autologous Hinge domain) significantly reduces the anti-Infliximab idiotypic (anti-I-Id) Ab binding to Pro-Infliximab by 108-fold as compared to Infliximab, and the TNF-α neutralizing ability of Pro-Infliximab can be completely restored after MMP-2/9 cleavage, thereby blocking TNF-α downstream signaling [100]. These data indicate that the pro-Ab strategy may prevent the neutralizing and cleaning effect of anti-idiotypic Abs on Ab drugs, prolong the half-life of Ab drugs and in the future may potentially become an alternative therapeutic option in patients, who had already developed Ab drug-specific anti-idiotypic Abs.

### Improving the masking efficacy of pro-antibody by computer modeling

The masking efficiency of different Ab locks, especially spatial-hindrance-based Ab locks, depends on the occupied site or interference range of the masking domain in front of antigen binding site of Ab drugs. The autologous hinge developed by Lu et al. has been proved to widely mask anti-TNF-α Ab (Infliximab), anti-IL-6 receptor Ab, anti-IL-1β Ab, anti-PD-1 Ab and anti-CTLA-4 Ab by 395-fold, 50.3-fold [100], 68-fold, 256-fold and 106-fold (data not shown), respectively. The coiled-coil domain suggested by Seattle Genetics. Inc. can broadly and efficiently block the antigen binding ability of anti-CD20 mAb (Rituximab), anti-HER2 mAb (Trastuzumab), anti-αVβ6 mAb (h15H3) and anti-mouse CD3 mAb (145-2C11) at least 80-fold, 470-fold, 290-fold and 1000-fold, respectively [176]. The LAP domain screened out by Chen et al. can also inhibit the antigen binding activity of anti-EGFR Ab (Cetuximab) or anti-TNF-α Ab (Infliximab) both by 1.86-fold [27]. However, even masking with the same Ab lock, there is inconsistent masking efficiency between different target mAbs. We reasonably think that there is still slight structural difference at the complementary-determining regions (CDRs) or N-terminal end of different mAbs despite the high structural conservation of Abs between different species, leading to the inconsistent masking effect of Ab locks on the binding activity of Ab drugs. In order to optimize the masking efficiency of the Ab lock to different mAbs, adjusting the linker sequence between the Ab lock and Ab drugs by amino acid addition, deletion or substitution, and combining with structure-based computational simulation (such as structure building by using Discovery Studio (San Diego, CA, USA) or molecular dynamic simulation by using Amber software) may assist scientists in rationally predicting and adjusting the length or bending angle of the connecting linker and optimizing the coverage rate of different Ab locks to obtain the highest masking efficiency for various Ab drugs.

## Conclusions

Monoclonal Abs continue to be the most rapidly growing and valuable drug field in the pharmaceutical industry due to their high specificity and affinity to target antigens and longer half-life than conventional drugs. However, there is still an urgent need to improve the selectivity of mAbs to distinguish target antigens at the disease site from normal healthy tissue, thereby reducing the severe adverse events caused by mechanism-of-action-related effects of mAbs and elevating its safety and therapeutic efficacy during systemic administration. The development of masking approaches to Ab drugs can improve the local reactivity of mAbs at the disease site, increase the therapeutic efficacy and safety of long-term treatment with mAbs in chronic diseases, and even serve as an alternative therapeutic option for preventing the functional interference of anti-idiotypic Abs. Masking approaches can also permit scientists to develop next generation Ab drugs for targets that have been hitherto undruggable and satisfy the unmet medical needs of mAb therapy.

## Data Availability

Not applicable.

## Abbreviations

TNF-α: Tumor necrosis factor α
CD: Cluster of differentiation
CTLA-4: Cytotoxic T-lymphocyte-associated protein 4
HER2: Human epidermal growth factor receptor 2
HER3: Human epidermal growth factor receptor 3
NK: Natural killer cell
Treg: Regulatory T cell
PML: Progressive multifocal leukoencephalopathy
CRS: Cytokine-released syndrome
Ab: Antibody
Ag: Antigen
IgG1: Immunoglobulin G1
CDR: Complementary-determining region
c-Met: Mesenchymal epithelial transition factor
VCAM-1: Vascular cell adhesion molecule 1
DM1: N2’-deacetyl-N2’-(3-mercapto-1-oxopropyl)-maytansine
HIV: Human immunodeficiency virus
EGFR: Epidermal growth factor receptor
MW: Molecular weight
LAP: Latency-associated peptide
dsFv: Disulfide-stabilized variable fragment
H: Heavy chain
L: Light chain
MMP: Matrix metalloprotease
ADAM: A disintegrin and metalloproteinase
uPA: Urokinase plasminogen activator
PD-1: Programmed cell death protein 1
PD-L1: Programmed death-ligand 1
VEGF-A: Vascular endothelial growth factor A
VEGFR2: Vascular endothelial growth factor receptor 2
IL: Interleukin
IL-6R: IL-6 receptor
RANKL: Receptor activator of nuclear factor kappa-Β ligand
BACE1: β-site amyloid β precursor protein cleaving enzyme 1
Aβ: Amyloid β
mAbs: Monoclonal antibodies
Pro-Ab: Pro-antibody
FDA: US Food and Drug Administration
CAGR: Compound annual growth rate
RA: Rheumatoid arthritis
JCV: John Cunningham virus
NHL: Non-Hodgkin’s lymphoma
EMA: European Medicines Agency
CLL: Chronic lymphocytic leukaemia
CIA: Collagen-induced arthritis
PK: Pharmacokinetics
CC: Coiled-coil
ADCs: Ab-drug conjugates
aDVD: Activatable dual variable domain
ICAM-1: Intercellular adhesion molecule 1
SPR: Surface plasmon resonance
PSCA: Prostate stem cell antigen
MT-SP1: Membrane type-serine protease 1
TMDD: Target-mediated drug disposition
scFv: Single chain Fv
sEGFRdIII: Soluble EGFR domain III
dsDNA: Double strand DNA
ssDNA: Single strand DNA
ECM: Extracellular matrix
ALL: Acute lymphoblastic leukemia
COPD: Chronic obstructed pulmonary disease
ADA: Anti-drug antibody
NF-κB: Nucleus factor kappa B
n.d.a.: No data available
